# Supplementary material for: Evaluation of Approaches for the Assessment of HER2 Expression in Breast Cancer by Radionuclide Imaging Using the Scaffold Protein [99mTc]Tc-ADAPT6
Source: Pharmaceutics. 2024 Mar 23;16(4):445. doi: 10.3390/pharmaceutics16040445 (PMC11053875; doi:10.3390/pharmaceutics16040445)
Supplement: Supplementary file 1 [file pharmaceutics-16-00445-s001.zip › pharmaceutics-2899164-supplementary.pdf]

Table S1. Eligibility criteria.

**Inclusion criteria**	**Exclusion criteria**
• A subject is over 18 years of age;<br>• Diagnosis of primary breast cancer with lymph node metastases;<br>• Availability of results from HER2 status test previously determined using a biopsy material from a primary tumour and metastatic LN, either HER2-positive, defined as a DAKO HercepTest™ score of 3+ or FISH positive or HER2-negative. defined as a DAKO HercepTest™ score of 0 or 1+ or else if 2+ then FISH negative;<br>• Haematological, liver and renal function test results within the normal limits;<br>• A negative pregnancy test was requested from all patients of childbearing potential and capability to undergo the diagnostic investigations planned in the study	• Any system therapy (chemo-/targeted therapy);<br>• A second, non-breast malignancy;<br>• Active current autoimmune disease or history of autoimmune disease;<br>• Active infection or history of severe infection within the previous 3 months (if clinically relevant at screening);<br>• Known positive HIV test or chronically active hepatitis B or C;<br>• Administration of other investigational medicinal products within 30 days of screening;<br>• Ongoing toxicity > grade 2 from previous standard or investigational therapies. according to the US National Cancer Institute.

Table S2. Accumulation [^{99m}Tc]Tc-ADAPT6 in primary breast tumours, reference organs and tumour-to-reference tissue ratios.

№	SUV_{max} (breast tumour)	SUV_{max} (Backg.)	Tumour-to-contralat. ratio	SUV_{max} (liver)	SUV_{max} (LDM)	SUV_{max} (spleen)	Tumour-to-liver ratio	Tumour-to-LDM ratio	Tumour-to-spleen ratio
HER2-positive tumours									
1	9.6	0.36	26.67	3.65	0.14	2.45	2.63	68.57	3.92
2	6.19	0.38	16.29	1.96	0.29	0.8	3.16	21.34	7.74
3	8.41	0.7	12	2.74	0.62	1.99	3.07	13.56	4.23
4	10.25	0.44	23.29	3.18	0.44	0.55	3.22	23.29	18.64
6	7.93	0.66	12	2.88	0.48	1.69	2.75	16.52	4.69
8	9.09	0.55	16.5	3.09	0.58	1.39	2.94	15.67	6.54
10	7.89	0.63	12.5	2.74	0.42	1.06	2.88	18.78	7.44
11	6.67	0.56	11.9	4.73	0.44	0.91	1.41	15.16	7.33
14	15.46	0.42	36.8	5.9	0.23	1.49	2.62	67.22	10.37
15	6.63	0.76	8.72	2	0.65	0.94	3.31	10.2	7.05
18	5.68	1.08	5.2	1.98	0.37	0.88	2.82	15.08	6.34
20	11.5	0.38	30.3	3.48	0.41	2.91	3.30	28.05	3.95
	8.8±2.7	0.57±0.21	17.7±9.54	3.2±1.2	0.42±0.15	1.4±0.7	2.8±0.51	26.1±20.1	7.3±4.02
HER2-negative tumours									
5	1.72	0.54	3.18	2	0.16	1.14	0.86	10.75	1.51
7	2.36	0.34	6.94	2.32	0.31	1.59	1.02	7.61	1.48
9	5.62	0.53	10.6	2.36	0.38	1.1	2.38	14.79	5.11
12	5.47	0.67	8.5	3.39	0.48	1.68	1.61	11.39	3.26
13	2.64	1.01	2.6	2.7	0.68	1.25	0.98	3.88	2.11
16	2.93	0.51	5.74	3.7	0.62	1.75	0.79	4.72	1.67
17	3.98	0.53	7.51	3.48	0.31	1.8	1.14	12.84	2.21
19	3.34	0.43	7.75	2.84	0.51	1.65	1.18	6.55	2.02
	3.5±1.4	0.6±0.2	6.6±2.7	2.8±0.6	0.43±0.2	1.5±0.3	1.2±0.5	9.1±3.9	2.4±1.2

LDM – latissimus dorsi muscle

Table S3. Accumulation [99mTc]Tc-ADAPT6 in metastatic axillary lymph nodes, reference organs and mALN-to-reference tissue ratios.

№	SUV_{max} (mALN)	SUV_{max} (Backg. ALN)	mALN -to-contralat. ratio	SUV_{max} (liver)	SUV_{max} (LDM)	SUV_{max} (spleen)	mALN -to-liver ratio	mALN -to-LDM ratio	mALN -to-spleen ratio
HER2-positive mALN									
1	14.64	0.37	39.56	3.65	0.14	2.45	4.01	104.57	5.98
2	4.7	0.22	21.36	1.96	0.29	0.8	2.39	16.21	5.88
3	4.34	0.29	14.96	2.74	0.62	1.99	1.58	7	2.18
4	6.52	0.11	59.27	3.18	0.44	0.55	2.05	14.82	11.85
6	2.88	0.21	13.7	2.88	0.48	1.69	6	1.70	1.04
8	14.57	0.38	38.3	3.09	0.58	1.39	4.71	25.12	10.48
10	8.62	0.08	107.75	2.74	0.42	1.06	3.15	20.52	8.13
11	6.04	0.64	10.4	4.73	0.44	0.91	1.28	13.73	6.64
14	12.29	0.48	25.6	5.9	0.23	1.49	2.083	53.43	8.25
15	6.92	0.45	15.38	2	0.65	0.94	3.46	10.65	7.37
19	3.82	0.31	12.3	1.98	0.37	0.88	1.34	7.49	2.31
20	16.65	0.36	46.25	3.48	0.41	2.91	4.78	40.61	5.72
	8.7±4.6	0.3±0.2	34.3±27.6	3.2±1.2	0.4±0.15	1.4±0.7	2.8±1.3	27.3±28.0	6.7±2.9
HER2-negative mALN									
5	1.31	0.12	10.9	2	0.16	1.14	0.66	8.19	1.15
7	1.95	0.27	7.2	2.32	0.31	1.59	0.84	6.29	1.22
9	4.1	0.26	15.76	2.36	0.38	1.1	1.74	10.79	3.73
12	1.84	0.26	7.07	3.39	0.48	1.68	0.54	3.83	1.1
13	2.06	0.36	5.7	2.7	0.68	1.25	0.76	3.03	1.65
16	2.54	0.35	7.25	3.7	0.62	1.75	0.68	4.1	1.45
17	3.13	0.44	7.11	3.48	0.31	1.8	0.9	10.1	1.74
18	5.68	0.3	18.9	2.84	0.51	1.65	2.87	15.35	6.45
	2.6±0.9	0.2±0.1	9.2±3.5	2.8±0.6	0.43±0.2	1.5±0.3	0.9±0.4	6.7±2.9	1.8±0.9

mALN – metastatic axillary lymph node; LDM – latissimus dorsi muscle

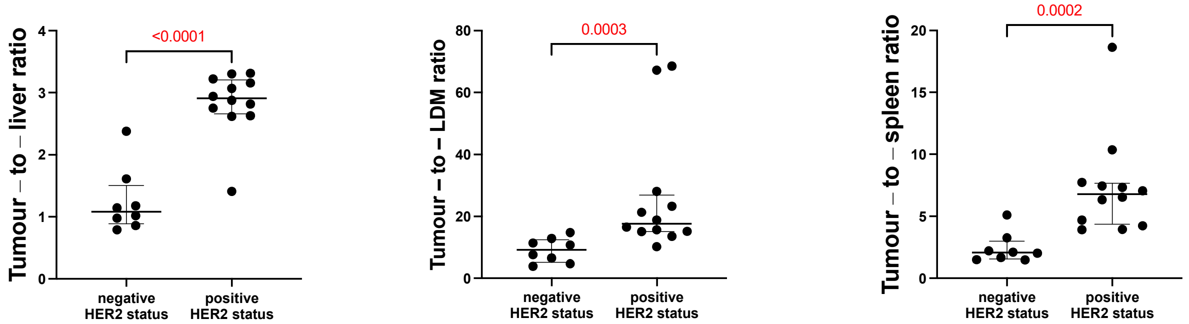

Figure S1. Tumour-to-liver, tumour-to-LDM and tumour-to-spleen ratios in HER2-positive and HER2-negative primary breast tumours. P values were determined using Mann–Whitney test.

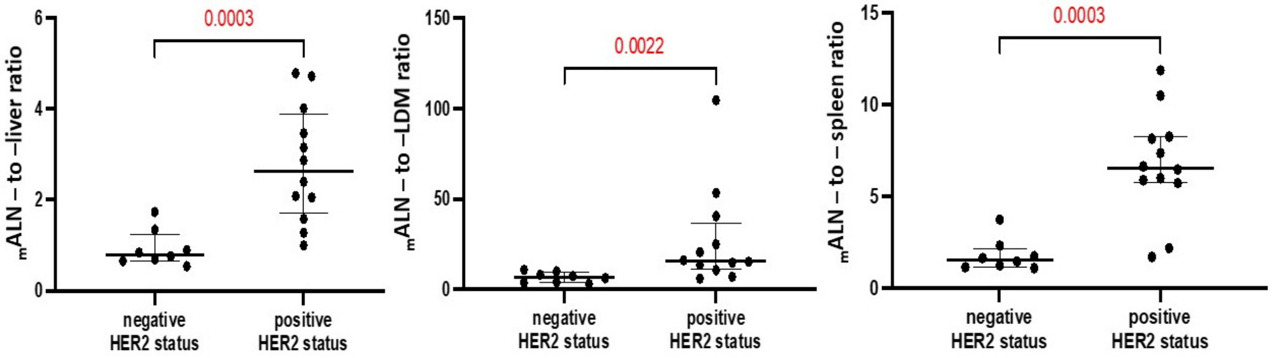

Figure S2. mALN-to-liver, mALN-to-LDM and mALN-to-spleen ratios in HER2-positive and HER2-negative metastatic axillary lymph nodes. P values were determined using Mann–Whitney test.

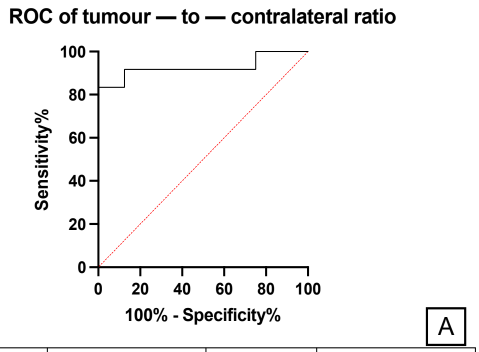

	Sensitivity%	95% CI	Specificity%	95% CI
> 8.610	91.67	64.61% to 99.57%	87.50	52.91% to 99.36%

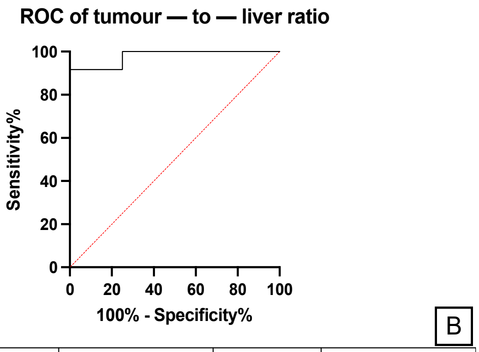

	Sensitivity%	95% CI	Specificity%	95% CI
> 2.501	91.67	64.61% to 99.57%	100.0	67.56% to 100.0%

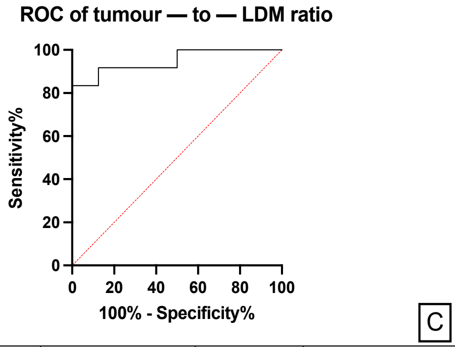

	Sensitivity%	95% CI	Specificity%	95% CI
> 14.94	83.33	55.20% to 97.04%	100.0	67.56% to 100.0%

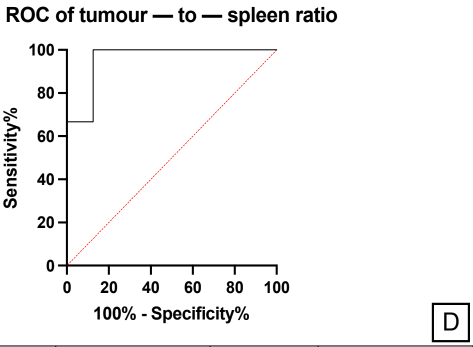

	Sensitivity%	95% CI	Specificity%	95% CI
> 3.590	100.0	75.75% to 100.0%	87.50	52.91% to 99.36%

Figure S3. The Receiver operating characteristic (ROC) curve analysis estimating the cut-off values of tumour-to-organ ratios for primary breast cancer. The reference tissues are (A) the contralateral site, (B) the liver, (C) LDM and (D) the spleen.

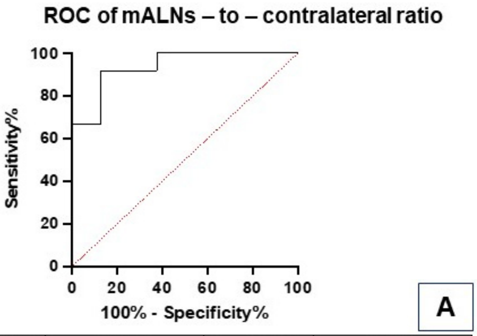

	Sensitivity%	95% CI	Specificity%	95% CI
> 13.00	91.67	64.61% to 99.57%	87.50	52.91% to 99.36%

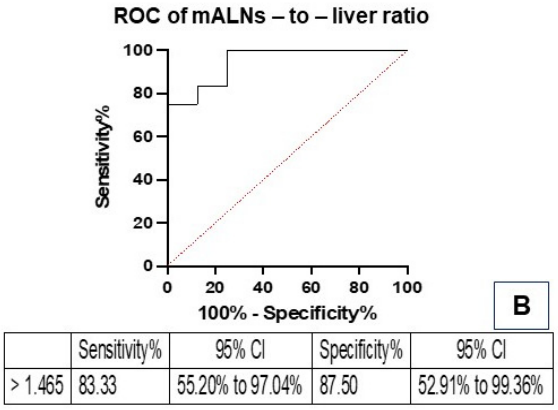

	Sensitivity%	95% CI	Specificity%	95% CI
> 1.465	83.33	55.20% to 97.04%	87.50	52.91% to 99.36%

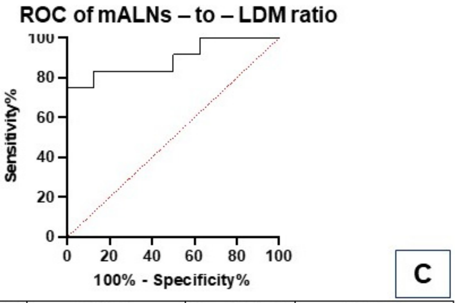

	Sensitivity%	95% CI	Specificity%	95% CI
> 10.37	83.33	55.20% to 97.04%	87.50	52.91% to 99.36%

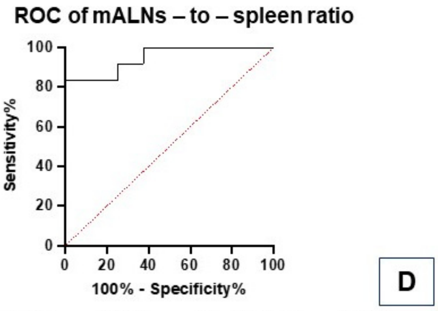

	Sensitivity%	95% CI	Specificity%	95% CI
> 4.724	83.33	55.20% to 97.04%	100.0	67.56% to 100.0%

Figure S4. The Receiver operating characteristic (ROC) curve analysis estimating the cut-off of tumour-to-organ ratios for metastatic axillary lymph nodes. The reference tissues are (A) the contralateral site, (B) the liver, (C) LDM and (D) the spleen
